# Do K_ATP_ channels have a role in immunity?

**DOI:** 10.3389/fimmu.2024.1484971

**Published:** 2024-11-28

**Authors:** Stefan Feske, Francesco Colucci, William A. Coetzee

**Affiliations:** ^1^ Department of Pathology, NYU Grossman School of Medicine, New York, NY, United States; ^2^ Department of Obstetrics and Gynecology, University of Cambridge, Cambridge, United Kingdom; ^3^ Department of Physiology & Neuroscience, NYU Grossman School of Medicine, New York, NY, United States; ^4^ Department of Biochemistry and Molecular Pharmacology, NYU Grossman School of Medicine, New York, NY, United States

**Keywords:** ion channels, K_ATP_ channels, immunity, K^+^, cytotoxic lymphocyte, NK cells, CD8+ T lymphocyte subsets

## Abstract

Ion channels, exchangers and pumps are expressed ubiquitously in cells from all phyla of life. In mammals, their role is best described in excitable cells, where they regulate the initiation and propagation of action potentials. There are over 70 different types of K^+^ channels subunits that contribute to these processes. In non-excitable cells, K^+^ channels set the resting membrane potential, which in turn drives the activity of other translocators. K^+^ channels also help maintain cell volume, influence cell proliferation and apoptosis and regulate Ca^2+^ signaling, which in turn is crucial for many cellular processes, including metabolism, secretion, and gene expression. K^+^ channels play crucial roles in the activation, proliferation and a variety of other functions in cells of the innate and adaptive immune system. The ATP-sensitive K^+^ (K_ATP_) channel has an established role in diverse cells, but its presence and function in immunity is scantly described. Public gene expression databases show that K_ATP_ channel subunits are highly expressed in NKT and NK cells, and that they are significantly upregulated after infection in CD8+ T cells and macrophages. We discuss these findings in the light of the available literature and propose a role for K_ATP_ channels in cytotoxicity of cells that are primed for a rapid immune response. Possible underlying molecular mechanisms are discussed.

## Ion channels in immune cells

Regardless of the cell type, all cells require ion translocators (channels, exchangers and pumps) to establish transmembrane ion concentration differences, which in turn drive multiple membrane transport systems to regulate many biological processes such as metabolism, pH regulation, Ca^2+^ homeostasis, transcriptional profiles and others. Additionally, these ion translocators establish a negative membrane potential inside cells that is a necessary feature for excitable and non-excitable cells alike. Although the field is not extensively developed in immunology, immune cells have a rich complement of ion translocators (1, 2). In T cells, B cells, monocytes, macrophages, dendritic cells and neutrophils, certain K^+^ channels (KCa3.1 and Kv1.3) have prominent roles. Other ion channels with key roles include Ca^2+^ entry channels (mainly ORAI1/STIM1), P2X receptors, and some members of the transient receptor potential (TRP) family (1). The involvement of ion channels in immunity has been reviewed (1–3) and is highlighted by inherited gene variants (channelopathies) in immunodeficiency disorders (4).

## K^+^ channels

There is a rich diversity of K^+^ channels in mammals. Several classes of K^+^ channels exist, each with specific functions and properties. These include voltage-gated K^+^ channels (Kv), which open or close in response to changes in membrane potential. Ca^2+^-activated K^+^ channels (KCa) open in response to the binding of intracellular Ca^2+^ ions, linking intracellular signaling to changes in membrane potential and Ca^2+^ homeostasis. Inward-rectifier K^+^ channels (Kir) and two-pore domain K^+^ channels (K2P) help to set the resting membrane potential, often in response to diverse signals (G-protein signaling, pH, arachidonic acids, etc.). There are over 70 protein K^+^ channels subunits, but the functional diversity is much higher since they are modulated by accessory subunits, alternative mRNA splicing, subunit assembly, and protein post-transcriptional modifications (5). The activity of K^+^ channels is regulated by changes in the membrane voltage, intracellular Ca^2+^ levels, the levels of PI (4, 5) P_2_ in the membrane, G-protein activity, pH, phosphorylation and dephosphorylation, palmitoylation, and many other events.

K^+^ channels have crucial roles in mammalian cells and contribute to many physiological processes. They are fundamental in setting and maintaining the resting membrane potential of cells. In excitable cells such as neurons and muscle cells, K^+^ channels are vital for repolarization the action potential and they participate in impulse propagation, thereby controlling neuronal function and influencing heart rate and rhythm. In smooth muscle, they regulate cell contraction and relaxation, and therefore blood flow. K^+^ channels are equally important in non-excitable cells, where their functions include the establishment of a negative membrane potential, which is turn is essential for diverse cell functions, such as controlling Ca^2+^ fluxes. K^+^ channels in epithelial cells are involved in the secretion and absorption processes. In the kidney, this process is crucial for the reabsorption of K^+^ and other ions, which is maintains the electrolyte balance and blood pressure. K^+^ channels also help maintain cell volume by regulating the flow of K^+^ and, consequently, water across the cell membrane in order to prevent cell swelling or shrinkage in response to osmotic changes. K^+^ channels can influence cell proliferation and apoptosis (programmed cell death). Overall, K^+^ channels are essential for numerous physiological processes in mammalian cells, playing key roles in maintaining cellular homeostasis and enabling complex cellular functions.

K^+^ channels have well-documented functions in immune cells (1, 6–9). In addition to their role in maintaining the resting membrane potential, they are crucial for various other processes. In T cells, specific K^+^ channels, such as the voltage-gated Kv1.3 and the Ca^2+^-activated KCa3.1 channels, play crucial roles in activation and proliferation. Kv1.3 and KCa3.1 channels are involved in setting the membrane potential (7). They also facilitate Ca^2+^ influx through Ca^2+^-release-activated calcium (CRAC) channels in T cells, B cells, and macrophages (10, 11), which is essential for processes such as T cell receptor (TCR) signaling and activation. K^+^ channels contribute to the effector functions of immune cells, such as the production and release of cytokines, chemokines, and other signaling molecules. By regulating membrane potential and Ca^2+^ signaling, K^+^ channels influence the activation of transcription factors and other signaling pathways that control gene expression related to immune responses. Other proposed roles include the process of chemotaxis, where immune cells move toward the site of infection or inflammation, and influencing the process of apoptosis. In addition to Kv1.3 and KCa3.1, the two-pore domain TASK1 and TASK3 K^+^ channels contribute to the background K^+^ conductance and help set the resting membrane potential (12). The TWIK2 and THIK-1 K^+^ channels, moreover, have been reported to regulate inflammasome activation (13, 14). In summary, K^+^ channels are essential for the proper functioning of immune cells, influencing their activation, proliferation, effector functions, migration, and apoptosis. Some K^+^ channels such as Kv1.3 and KCa3.1 are being targeted to treat immune diseases and neuroinflammation (15–17).

## The K_ATP_ channel

The ATP-sensitive K^+^ (K_ATP_) channel is unique among all of the different types of K^+^ channels discussed above since it directly couples intracellular energy metabolism to K^+^ flux and subsequent cellular events. It does so because the K_ATP_ channel opening is regulated by the intracellular levels of ATP, ADP and AMP (18). In the “normal” well-oxygenated cellular state, intracellular ATP levels are high and the levels of ADP and AMP are low due to the action of adenylate kinase. Under these conditions, ATP directly binds to the channel and causes the K_ATP_ channel to be in the closed state. By contrast, when intracellular ATP levels fall and ADP levels increase (such as during hypoxia or in a metabolically stressed state), K_ATP_ channel opening is favored. Thus, via effects on the K_ATP_ channel, changes in the intracellular metabolic state can regulate K^+^ flux, membrane potential and (in excitable cells) changes in the action potential duration.

### Roles of K_ATP_ channels

**Figure 1:**
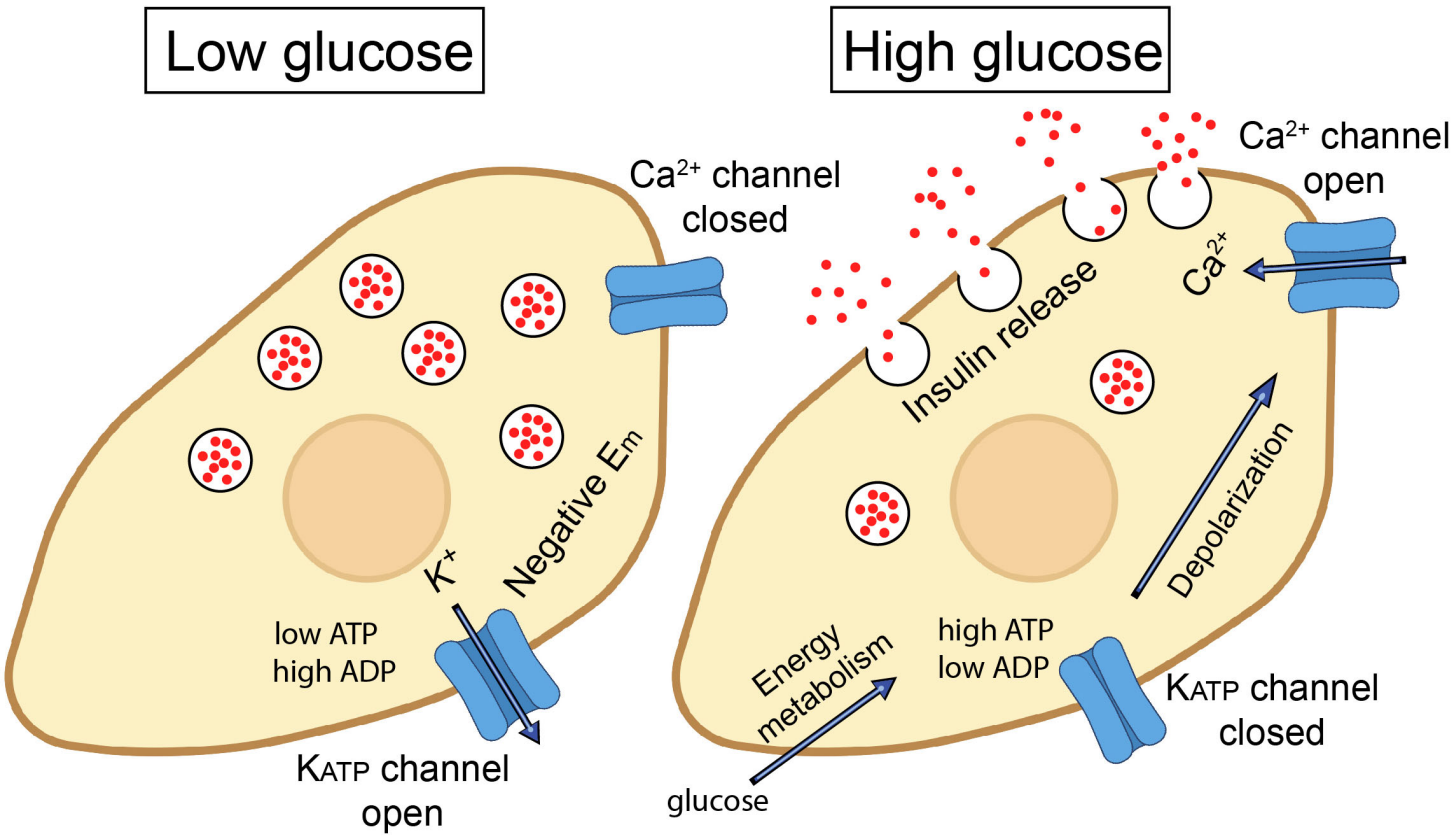
Role of ATP-sensitive K+ (K_ATP_) channels in insulin secretion from pancreatic β cells. In the resting state (at low plasma glucose levels), K_ATP_ channels are open. K^+^ flow efflux from the β cell maintains a hyperpolarized state (negative membrane potential; E_m_). When blood glucose levels rise, glucose enters through GLUT2 transporters and is metabolized via glycolysis and the citric acid cycle, leading to an increase in intracellular ATP. The increased ATP/ADP ratio closes the K_ATP_ channels, leading to membrane depolarization. This, in turn, triggers the opening of voltage-gated Ca^2+^ channels and influx of Ca^2+^ ions. Elevated intracellular Ca^2+^ triggers the exocytosis of insulin-containing vesicles from the β cells, releasing insulin into the bloodstream, which helps to lower blood glucose levels by promoting the uptake of glucose into tissues, such as muscle and adipose tissue.

K_ATP_ channels are expressed almost ubiquitously, with pronounced roles in tissues such as the pancreas, vascular smooth muscle, the heart, and the nervous system. The K_ATP_ channels have diverse roles in many cell types throughout the body. These include the regulation of insulin secretion in pancreatic β cells after a meal, protection of cardiac cells during metabolic stress such as ischemia, regulation of the vascular tone and blood flow, neuroprotection during periods of metabolic stress such as during stroke or hypoxia, and their contribution to the regulation of skeletal muscle contraction and energy efficiency, especially under conditions of metabolic stress. Overall, K_ATP_ channels are key for linking cellular metabolic states to membrane excitability and they function across a wide range of tissues. For more details, please refer to recent reviews on K_ATP_ channels (19–23). An example of the function of the K_ATP_ channel in pancreatic β cells of the islet of Langerhans is illustrated in **Figure 1**. Under fasting conditions, the metabolic state of the β cell is low and the K_ATP_ channel is in the open state. The K^+^ flux via the K_ATP_ channels from the cell causes the membrane potential to be negative. After a meal, when the plasma glucose levels rise, glucose is metabolized, causes an increase in cytosolic ATP levels and lower cytosolic ADP levels. K_ATP_ channels are blocked under these conditions, which depolarizes the membrane potential, leading to action potential firing. This, in turn, opens voltage-gated channels such as Ca^2+^ channels. The resulting Ca^2+^ entry, and Ca^2+^ release from intracellular stores, elevates cytosolic Ca^2+^ levels that triggers insulin-containing vesicles to dock to the plasma membrane and release their content from the β cells (19). This is an example of where K_ATP_ channels link intracellular energy metabolism to secretory processes. K_ATP_ channels can also cause secretion of neurotransmitters and other intracellular cargo. A major role of K_ATP_ channels in excitable cells, such as cardiac and skeletal muscle and neurons, is to couple intracellular energy metabolism to changes in membrane potential, action potential duration and to prevent intracellular Ca^2+^ overload (18). These events protect the cells against stress events, such as ischemia and hypoxia. A recent study demonstrated that ATP levels change measurably during each heartbeat, and that K_ATP_ channels open under physiological conditions in the heart (24).

### K_ATP_ channel subunits

There are several different subtypes of K_ATP_ channels in different tissues (25). Although they largely have the same role to couple intracellular energy metabolism to excitability, these K_ATP_ channel subtypes differ from each other in terms of their sensitivities to intracellular nucleotides, their biophysical properties, and pharmacological sensitivities (25, 26). The single channel conductance of K_ATP_ channels in pancreatic β-cells and cardiomyocytes, for example, is similar (~85 pS), but the pancreatic channel has a higher sensitivity to some openers (e.g. diazoxide) and blockers (e.g. glibenclamide) than the cardiac channel. Conversely, the sensitivities of the smooth muscle and pancreatic K_ATP_ channels to these two agents are similar, but they have different biophysical properties, such as the single channel conductance (18, 27, 28). These differences are due to unique and specific K_ATP_ channel subunit combinations in different tissues.

K_ATP_ channel subunits are encoded by four genes. *KCNJ8* and *KCNJ11 respectively* encode the Kir6.1 and Kir6.2 subunits. To form a functional channel, they assemble with one of two types of sulphonylurea receptors SUR1 or SUR2, which respectively are products of the *ABCC8* and *ABCC9* genes. There are several SUR1 and SUR2 splice variants (29), but the two variants most commonly studied are SUR2A and SUR2B, which differ from each other in the distal 42 amino acids of the C-terminus. Kir6.x and SURx subunits coassemble in specific combinations in a tissue-specific manner to give rise to the various K_ATP_ channel subtypes (**Table 1**).

**Table 1:** Diversity of K_ATP_ channel subunits and genes.

Tissue	Subunits	Pinacidil	Glibenclamide
Pancreatic β-cells	Kir6.2/SUR1	> 30 µM	0.87 nM
Cardiac and skeletal myocytes	Kir6.2/SUR2A	17 µM	9.9 nM
Vascular smooth muscle cells, endothelium and pericytes	Kir6.1/SUR2B	1 µM	21 nM

The IC_50_ values for pinacidil and glibenclamide are from Dyhring et al., 2023 (30).

### Structural aspects of K_ATP_ channels

**Figure 2:**
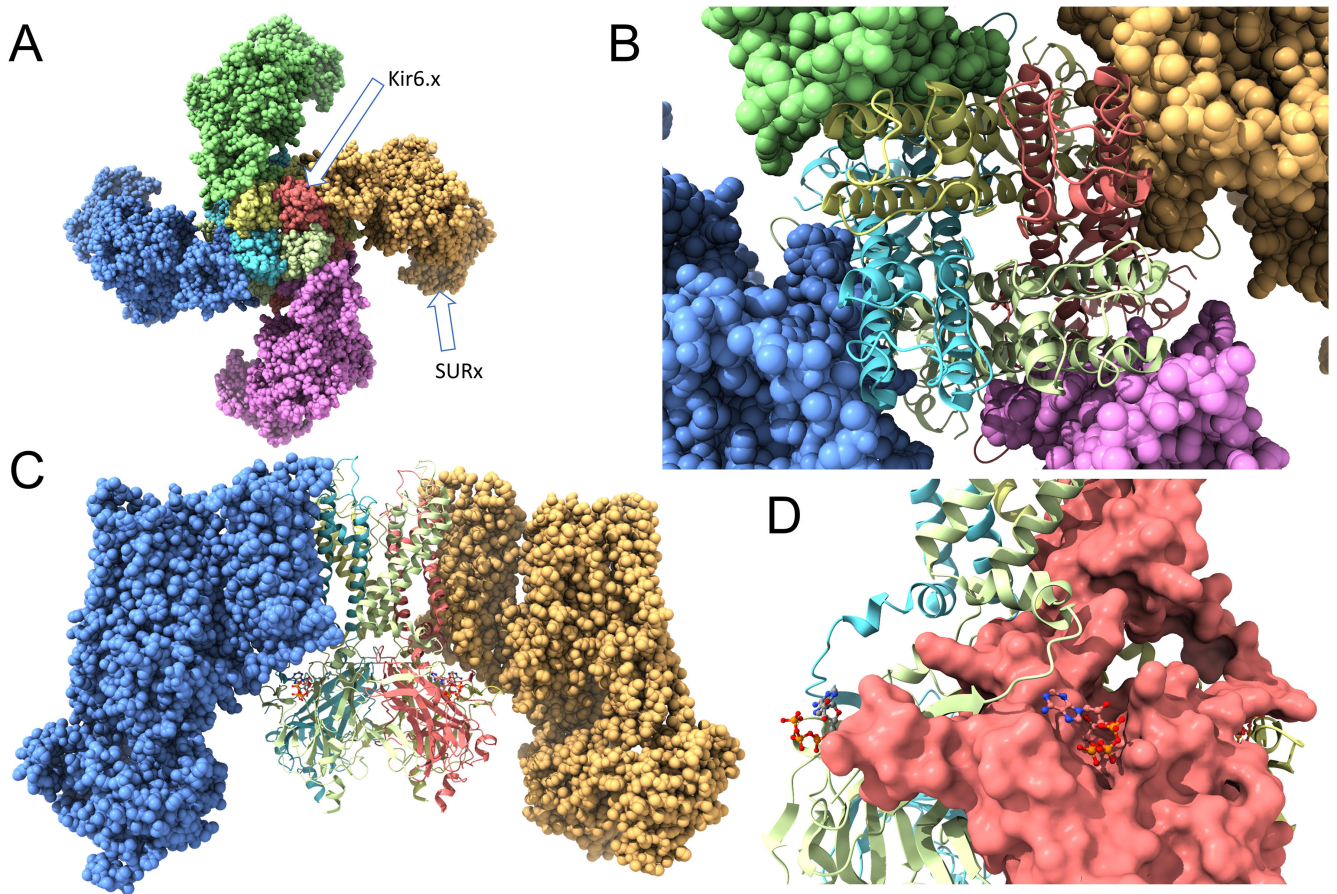
Structure of the ATP-sensitive K^+^ (K_ATP_) channel. **(A)** A view from the extracellular side. Four Kir6.x subunits form a membrane spanning central pore, with a K^+^ selectivity filter, that allows K^+^ to move through the membrane. They are surrounded by four SURx subunits that modulate the channel activity and, often, serve as binding sites for K_ATP_ channel openers and blockers. **(B)** The same structure as in **(A)**, but the Kir6.x subunits are shown in ribbon style to depict the M1 and M2 transmembrane domains of each subunit. **(C)** A view of the K_ATP_ channel perpendicular with the membrane. Two of the SURx subunits are hidden for clarity. This image depicts the Kir6.x M1 and M2 domains, and the intracellular N- and C-termini. **(D)** The solvent-accessible surfaces of the Kir6.x subunits are depicted. Also shown are the ATP molecules, demonstrating binding of ATP to the intracellular Kir6.2 C-terminus of one subunit, and with an N-terminal region of an adjacent Kir6.x subunit. This depiction was created with ChimeraX using PDB #6BAA as template.

Overall, K_ATP_ channels are hetero-octameric complexes (**Figure 2**). They are composed of four Kir6.x subunits that form the K^+^-selective pore through the membrane. In addition, the structure contains four SURx subunits, that regulate the channel’s activity. Each Kir6.x subunit has two transmembrane helices (M1 and M2), with a pore-forming loop (H5) between them that lines the ion conduction pathway. The four Kir6.x subunits come together to form the central pore of the channel. This tetrameric arrangement is similar to other inwardly rectifying K^+^ channels. The SUR subunits (SUR1, SUR2A, or SUR2B) belong to the ATP-binding cassette (ABC) transporter family. Each SUR subunit has 17 transmembrane helices organized into three transmembrane domains (TMD0, TMD1 and TMD2) and two intracellular nucleotide-binding domains (NBD1 and NBD2). The vast majority of pharmacological agents (e.g., sulfonylureas and K_ATP_ channel openers) bind to SURx, which in turn couples with Kir6x to alter channel opening. Intracellular ATP binds to the Kir6.x subunits, leading to channel closure. This is crucial for the channel’s ability to link metabolic state to membrane potential. Binding of intracellular ADP to SURx’s nucleotide binding domains (NBDs) promotes channel opening, counteracting the blocking effect of ATP on Kir6.x.

### Pharmacology of K_ATP_ channels

The pancreatic β-cell K_ATP_ channel is comprised of Kir6.2/SUR1 subunits and is more sensitive to opening by VU0071063 and block by gliclazide compared to the other channels. Smooth muscle K_ATP_ channels, composed of Kir6.1/SUR2B, are preferentially opened by pinacidil and blocked by PNU-37883A. The Kir6.2/SUR2A cardiac K_ATP_ channel is preferentially activated by CL-705G (30, 31). Apart from these few exceptions, however, there is a large overlap in the sensitivities of the different K_ATP_ channel subtypes to most K_ATP_ channel openers and blockers (18, 26). Glibenclamide is a second-generation sulfonylurea and is commonly used for treating type II diabetes mellitus. It has a high affinity for pancreatic receptors (SUR1) and is a potent insulin secretagogue.

## Clinical evidence for a possible involvement of K_ATP_ channels in immunity

There are no controlled clinical trials to specifically study the involvement of K_ATP_ channels in immunity and infection. There are, however, a few incidental reports mentioning a possible role in immunity in diabetic patients treated with sulphonylureas (K_ATP_ channel blockers). For example, the effect of glibenclamide on cytokine secretion was investigated with granulocytes from diabetic patients, to determine whether K_ATP_ channels contribute to patient susceptibility to bacterial infection (32). Indeed, glibenclamide reduced secretion of the inflammatory cytokines interleukin (IL)-1β and IL-8 when granulocytes were exposed to *B. pseudomallei*. Moreover, the IL-1β and IL-8 production by granulocytes obtained from diabetic subjects treated with glibenclamide was similarly decreased, demonstrating that glibenclamide affects cytokine secretion at therapeutic levels in diabetic patients. The same group investigated glibenclamide in the context of tuberculosis (33). Purified monocytes from non-diabetic and diabetic individuals were infected with *M. tuberculosis* (Mtb). Monocytes from diabetes patients treated with glibenclamide secreted less IL-1β and IL-8 when exposed to Mtb. Additionally, these responses also occurred when monocytes from non-diabetic individuals were pre-treated with glibenclamide *in vitro*. A direct effect of glibenclamide was also demonstrated in human whole blood *ex vivo*. Glibenclamide reduced LPS-induced release of IL-1β and TNF-α in a concentration-dependent manner (34). Finally, another group examined a cohort of 1,160 patients with Gram-negative sepsis caused by *Burkholderia pseudomallei*. Of these, 35% of the patients were diabetic. Survival was significantly greater in diabetic patients taking glibenclamide when compared to either the nondiabetic patients or to the diabetic patients not taking the sulphonylureas (35). Overall, therefore, these limited studies suggest that glibenclamide possesses beneficial anti-inflammatory actions in humans. It remains to be established whether the apparent anti-inflammatory action of glibenclamide is due to actions on K_ATP_ channels in the clinical setting, or whether the drug has possible non-K_ATP_ channel targets.

## K_ATP_ channel subunit expression in immune cells

There is a paucity of published data regarding K_ATP_ channel expression in immune cells. We therefore turned our attention to information available in publicly accessible databases. Specifically, we queried the ImmGen (Immunological Genome Project) database which is a gene-expression database for all characterized immune cells of C57BL/6 mice. Overall, of the four K_ATP_ channel subunit genes, expression appears to be largely restricted to *Kcnj8* and *Abcc9*. The expression of *Abcc8* (SUR1) and *Kcnj11* (Kir6.2) was very low in all immune cells in the database - these will not be further considered. *Kcnj8* and *Abcc9* are expressed in cells of both the innate and adaptive immune system. They are expressed in macrophages and certain types of CD8+ T cells after viral induction, and are constitutively expressed in NKT cells and mature populations of NK cells. Because *Kcnj8* and *Abcc9* expression matches those cells that have developed or innate functions to rapidly respond to infections or immune stressors, K_ATP_ channels may have a role in responses to these stressors.

**Figure 3:**
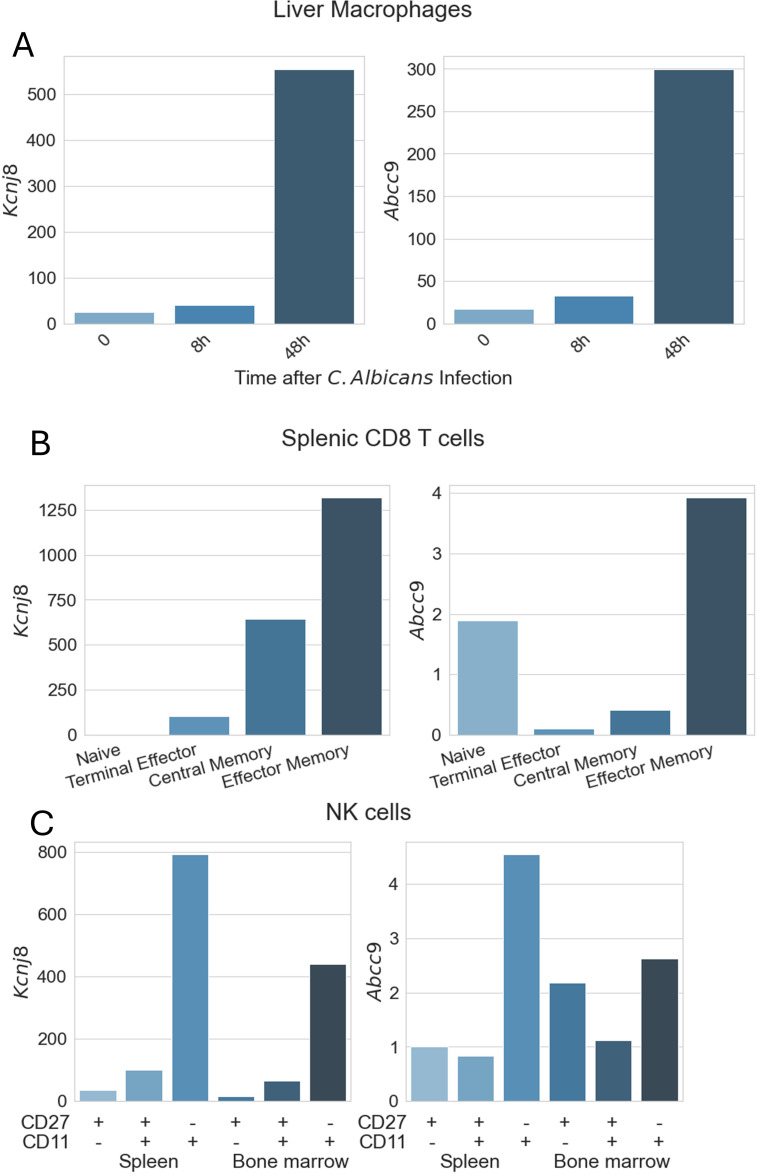
Expression of K_ATP_ channel subunits in immune cells. Data are from the ImmGen (Immunological Genome Project) database. Expression of *Abcc8* (SUR1) and *Kcnj11* (Kir6.2) was low in all immune cells present in the database. Shown are expression values for *Kcnj8* (Kir6.1) and *Abcc9* (SUR2) obtained with the Gene Skyline RNA Seq data. **(A)** Macrophage expression was obtained using ImmGen MNP OpenSource, selecting all macrophages as the reference cell type. For this graph, we filtered out liver macrophages of 8-week-old C57BL/6 mice (CD45+CD64+F4/80+MHCII+CD169+CD11blo F4/80hi.Lv) with data provided by the Lionakis lab. **(B)** CD8+ T cell data were obtained from ImmGen ULI RNAseq, selecting αβT Cells as the reference population. Data filtered out are splenic cells from C57BL/6 mice, with data provided by the Goldrath Lab. **(C)** NK cell data were obtained as for **(B)**, but selecting Innate Lymphocytes as cell type. Shown are splenic and bone marrow NK cells from C57BL/6mice, with data provided by the Lanier lab.

Of all the different types of macrophages (from different tissues) expression of *Kcnj8* (Kir6.1) and *Abcc9* (SUR2) was uniformly relatively low. A striking exception is that *Kcnj8* and *Abcc9* expression is strongly upregulated in liver macrophages after 8-48 h infection with *C. Albicans* (**Figure 3A**). There is other experimental evidence that K_ATP_ channel subunits are in fact upregulated in macrophages present in atherosclerotic plaques that correlates with increased inflammation (36). Moreover, blocking K_ATP_ channels with glibenclamide mitigates polarization of macrophages to the pro-inflammatory state, while opening K_ATP_ channels with pinacidil have the opposite effect (37). Thus, K_ATP_ channels may have a role in macrophages after infection, however this needs further exploration.

K_ATP_ channel gene expression in monocytes, dendritic cells, granulocytes, mast cells, basophils, and eosinophils were generally low (not illustrated). Likewise, *Kcnj8* and *Abcc9* expression in CD4+ T cells and naïve CD8+ T cells was low, with the highest expression in splenic NKT cells. *Kcnj8* and *Abcc9* expression was significantly upregulated in CD8+ T cells after lymphocytic choriomeningitis virus (LCMV) infection (**Figure 3B**). Of note, naive CD8+ T cells (mature cells that have not yet encountered their specific antigen) have low *Kcnj8* and *Abcc9* expression. Highly differentiated terminal effector CD8+ T cells at the end stage of differentiation, that produce large amounts of cytokines like IFN-γ and TNF, had moderately higher expression of these K_ATP_ channel genes. Central memory CD8+ T cells (T_CM_), with the potential for rapid proliferation upon re-exposure to specific antigens and their role in long-term immune surveillance, had even higher expression (particularly of *Kcnj8*). By far the highest *Kcnj8* and *Abcc9* expression was in effector memory CD8+ T cells (T_EM_). Memory CD8+ T cells can rapidly exert effector functions like cytokine production and cytotoxicity upon encountering their specific antigen.

**Figure 4:**
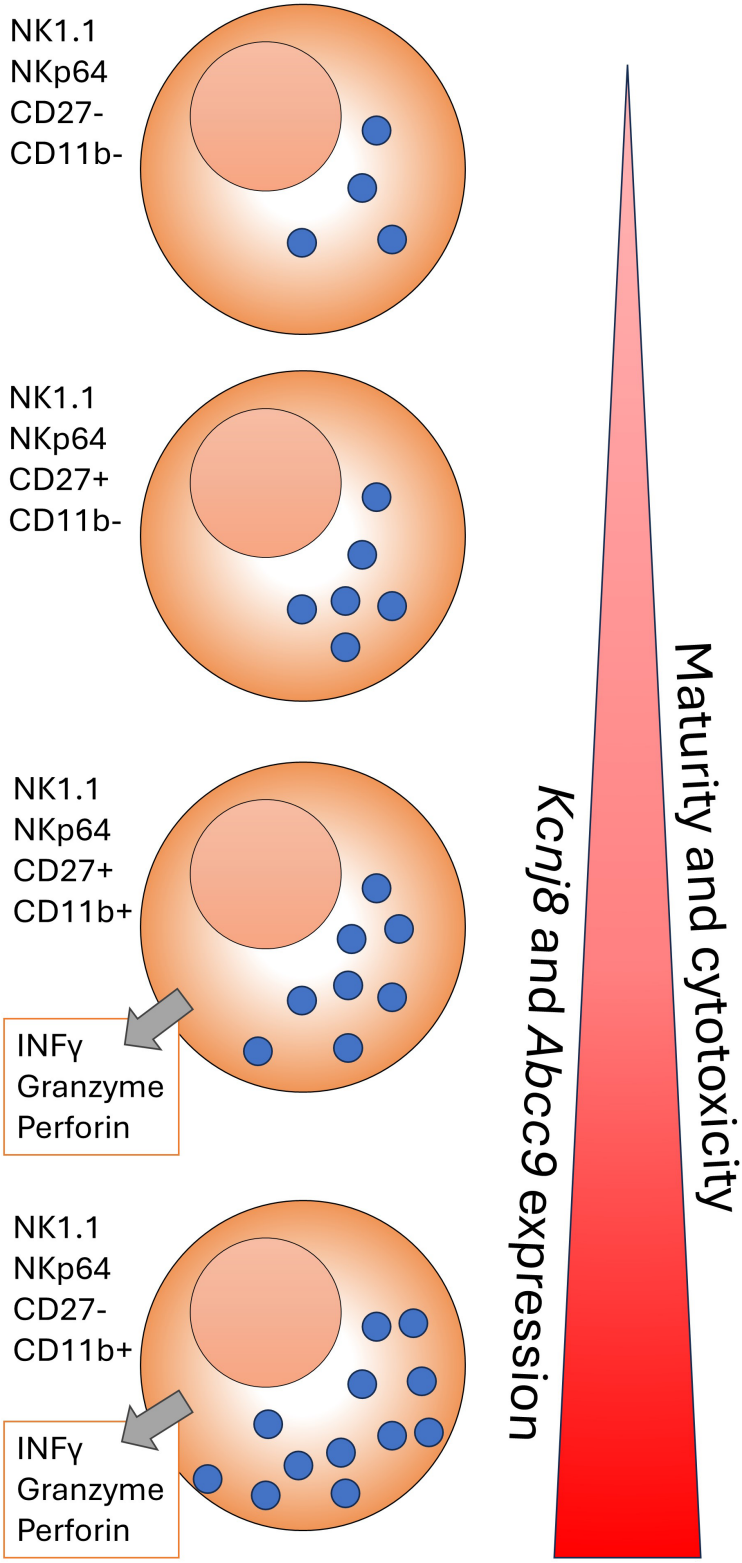
A cartoon representation of NK cell development in the C57BL/6mouse. NK cells form in the bone marrow and migrate to the periphery. The NK cells are marked by NK1.1, NKp46, and other markers. As maturity progresses, NK cells transition through CD27-CD11b-, CD27+CD11b-;, CD27+CD11b+ and CD27-CD11b+ subsets, becoming increasingly become more cytotoxic. The cytotoxicity can take the form of INFγ release and/or cytolysis of target cells via release of perforin and granzyme in the synaptic cleft that is formed between the NK cell and a target cell. This figure is based on a wall chart image created by Eric Vivier and Sophie Ugolini (https://www.stemcell.com/media/files/wallchart/WA10012-Natural_Killer_Cells.pdf.

Another cell type that is primed for quick responses are NK cells, which also express high levels of *Kcnj8* and *Abcc9* (**Figure 3C**). Interestingly, *Kcnj8* and *Abcc9* expression in both the spleen and bone marrow appears to track NK cell maturity, marked by the surface expression of CD27 and CD11b (38, 39) (**Figure 4**). There are preliminary reports demonstrating that Kcnj8 may have a role in NK cell function (40).

We reiterate that these data are all from mice and expression of K_ATP_ channel subunits in human immune cells is currently largely unknown.

## Genetic evidence in mice for a role of K_ATP_ channels in immunity

### Kcnj8 deficiency in mice leads to susceptibility to infection

There are genetic studies demonstrating that K_ATP_ channels have a role in immunity. One of the first studies to suggest this came from an observation that mice deficient of *Kcnj8* (Kir6.1) had an exaggerated susceptibility to challenge with lipopolysaccharide (LPS) since the survival of the knockout mice was greatly impaired (41). A subsequent random mutagenesis screen in mice with N-ethyl-N-nitrosourea (ENU) found several mutations that caused a profound susceptibility to infection with cytomegalovirus (MCMV), a double stranded DNA virus (42, 43). One of these, the *MayDay* mutant, showed significantly enhanced lethality 2 to 3 days after MCMV infection (42). Death occurred before high viral titers were achieved. The peak of cytokine response, measured 3 days post-inoculation, was minimally affected. This strongly raises the possibility that proinflammatory cytokines, rather than any MCMV cytopathic effects, might account for their susceptibility. The *MayDay* mutant mice additionally had a ~20,000-fold sensitization to the gram-negative bacterial component LPS which activates the toll-like receptor (TLR)4. *MayDay* mice were also hypersensitive to the lethal effects of poly(I:C) and CpG DNA, which activate TLR3 and TLR9 receptors, respectively. The *MayDay* mutation was mapped to a deletion of both exons in *Kcnj8*, essentially leading to a *Kcnj8* knockout. Interestingly, the *MayDay* mice succumbed to infection by *Listeria monocytogenes* administered at doses sublethal to wild-type mice, but not to infection by vesicular stomatitis virus (VSV). These data make a very strong case for a role of *Kcnj8* in immunity in mice. The questions to be addressed are the nature of the cell type(s) and the mechanism(s) involved. The potential confounding factors in studies using global Kcnj8 knockout mice can be addressed by generating mice deficient of Kcnj8 (or Abcc9) in specific immune cells, such as NK cells, CD8^+^ T cells or macrophages.

### Differences in pathogen susceptibility?

It is of interest that the *MayDay* mice were susceptible to *Listeria monocytogenes*, but not to infection by VSV. The former is a gram-positive, facultatively anaerobic foodborne bacterium, whereas VSV is an enveloped, negative-strand RNA virus belonging to the *Rhabdoviridae* family. The differential susceptibility of the *MayDay* mouse to these two infections could be attributed to several mechanisms related to the immune response or pathogen-specific virulence. Effective clearance of *Listeria* involves the innate immune system, particularly phagocytic cells like macrophages and neutrophils, which recognize and destroy the bacteria, whereas the innate immune response to VSV relies heavily on type I interferon (IFN) production and the activity of NK cells. The adaptive immune response, especially CD8+ cytotoxic T cells, plays a crucial role in clearing *Listeria* infections by killing infected cells. VSV clearance also involves adaptive immunity, particularly B-cell mediated antibody responses and CD8+ T cells. *Listeria* can survive and replicate within host cells, particularly macrophages. Mutations in genes involved in intracellular killing mechanisms (e.g., autophagy, phagosome-lysosome fusion) could make the mouse more susceptible to *Listeria*, which relies on evading intracellular defenses. Moreover, *Listeria* utilizes actin-based motility to spread from cell to cell. If the mutation affects cellular cytoskeletal dynamics or cell junctions, it might facilitate *Listeria*’s cell-to-cell spread and pathogenesis. VSV primarily infects cells through endocytosis and replicates in the cytoplasm. If *Kcnj8* does not impair the cellular processes involved in viral entry, replication, or egress, VSV infection might not be significantly affected. A possible role for pattern recognition receptors (PRRs) is also possible. For example, toll-like receptors (TLRs) and NOD-like receptors (NLRs) are crucial for recognizing bacterial components and initiating immune responses. If *Kcnj8* affects these receptors or their downstream signaling pathways (e.g., NF-κB or MAPK), it could impair the recognition and response to *Listeria*. Viral sensors such as RIG-I and MDA5 recognize viral RNA and initiate antiviral responses. If these pathways remain functional despite the loss of *Kcnj8*, then VSV replication might still be effectively controlled.

The role of *Kcnj8* in immune response to *Listeria monocytogenes* may potentially involve alterations in phagocytic cell function. For example, K_ATP_ channels may be involved in the regulation of macrophage and neutrophil functions, including the respiratory burst, which generates reactive oxygen species (ROS) crucial for killing intracellular pathogens like *Listeria*. A deficit of *Kcnj8* might impair the ability of these cells to produce ROS, leading to decreased bacterial killing. Proper function of K_ATP_ channels may also be necessary for the cytoskeletal rearrangements required during phagocytosis. Impaired function could lead to reduced uptake and destruction of *Listeria*. K_ATP_ channels may modulate the production of pro-inflammatory cytokines (e.g., IL-1β, TNF-α) in response to bacterial infections. A mutation in *Kcnj8* could lead to a dysregulated cytokine response, impairing the host’s ability to control *Listeria* infection.

### What cell type is responsible?

In the *MayDay* mutant mouse study, bone marrow transplantation was performed after irradiation (42). Five *MayDay* mice that had stable engraftment of wild type bone marrow and were allowed an 8-week recovery period. Two of the five engrafted mayday mice died within 36 h upon infection with MCMV. By contrast, none of the 13 irradiated Ly5.1+ wild type mice reconstituted with *MayDay* bone marrow died after infection with the same dose of MCMV. At face value, these results imply that the increased MCMV susceptibility is not due to defects in hematopoietic cells. It should be noted, however, that mice with global *Kcnj8* deficiency have a high postnatal mortally rate, are easily stressed, and can succumb suddenly from cardiac arrhythmias due to relatively minor stresses (44). About half of the *MayDay* mutant mice (4/9 mice) succumbed within 24 h post-irradiation (42), most likely due to the stress involved in the procedure. The stresses involved with bone marrow transplantation may also partly account for the high mortality in the *MayDay* mutant mice after MCMV infection. While this bone marrow transplantation experiment was necessary, the outcome should therefore be interpreted with caution. Experiments with conditional knockout of *Kcnj8* in specific immune cells are needed to resolve this question.

It is possible that extrahematopoietic cell type(s) contribute to confer the *Kcnj8* deficiency-related susceptibility to MCMV and LPS. If so, the nature of these cells is currently unknown and should be actively investigated. Since *Kcnj8* is expressed in the vasculature (as a Kir6.1/SUR2B complex; see **Table 1**), the most parsimonious explanation is that alterations in blood flow are involved. Indeed, a reduction in blood pressure was observed with LPS in the study that described the enhanced susceptibility to LPS in *Kcnj8* deficient mice (41). Moreover, the elevated TNF-α levels associated with LPS administration led to a doubling of the coronary flow rate, which was not observed with *Kcnj8* deficiency or K_ATP_ channel block by glibenclamide. It is possible, therefore, that the susceptibility to MCMV and LPS in the *Kcnj8* deficient mice or the *MayDay* mutant is attributed in part to a lack of vasodilatory responsiveness of coronary vessels in response to cytokines and/or metabolic stress. However, loss-of function of K_ATP_ channels in *Drosophila* (which express two Kir6.x subunits and a dSUR subunit) also indicate a role for K_ATP_ channels in anti-viral pathways in the fly (42, 45). The *Drosophila* myocardium lacks a coronary vascular system and the possibility should therefore be considered that extrahematopoietic cells that are not involved in coronary blood flow may be involved.

Cell types that express *Kcnj8* (other than those mentioned above) include lymphatic smooth muscle (46), brain microglia (47), and pericytes (48, 49). Microglia has a known role in immune surveillance and response in the brain. They can phagocytose (engulf and digest) pathogens, dead cells, and debris, thus maintaining CNS homeostasis. Microglia also have a prominent role in neuroinflammation. In response to injury or disease, microglia become activated and release cytokines and chemokines, which mediate inflammation. In addition to the role of pericytes to regulate blood flow in the heart and brain (49–51), pericytes also have immune functions. Pericytes respond to a range of pro-inflammatory stimuli and can sense danger signals by pattern-recognition receptors, contributing to the onset of innate immune responses (52). Pericytes overexpress adhesion molecules such as ICAM-1 and VCAM-1 involved in the control of immune cell trafficking across vessel walls. In response to pro-inflammatory stimuli, pericytes secrete a range of chemokines and cytokines, including IFN-γ, TNF-α, IL-1β, and IL-6 (52–54). Thus, pericytes are sentinels of the innate immune system and interact directly with several types of immune cells. Due to their unique localization at the interface between blood and parenchyma, pericytes are uniquely positioned to act as the first responders when sensing changes in the environment such as hypoxia, inflammation, pathogens. There is therefore a strong possibility that *Kcnj8* may participate in immunity by having a role in cell types such as microglia and/or pericytes.

## Possible molecular mechanisms

Even though there is a possibility that the anti-inflammatory role of *Kcnj8* against viral infection or LPS may be extrahematopoietic, effects within subsets of immune cells cannot be excluded given the expression pattern of *Kcnj8* and *Abcc9* in certain populations of immune cells, and the possible roles for K_ATP_ channels in macrophages (36, 37) and NK cells (40). Functions of *Kcnj8* and *Abcc9* need to be explored in NKT cells, NK cells, and macrophages and CD8+ T cells following infection. These functions may include regulation of cytokine release and/or cytolysis due to the release of cytotoxic molecules (perforin and granzyme) into the synaptic cleft.

**Figure 5:**
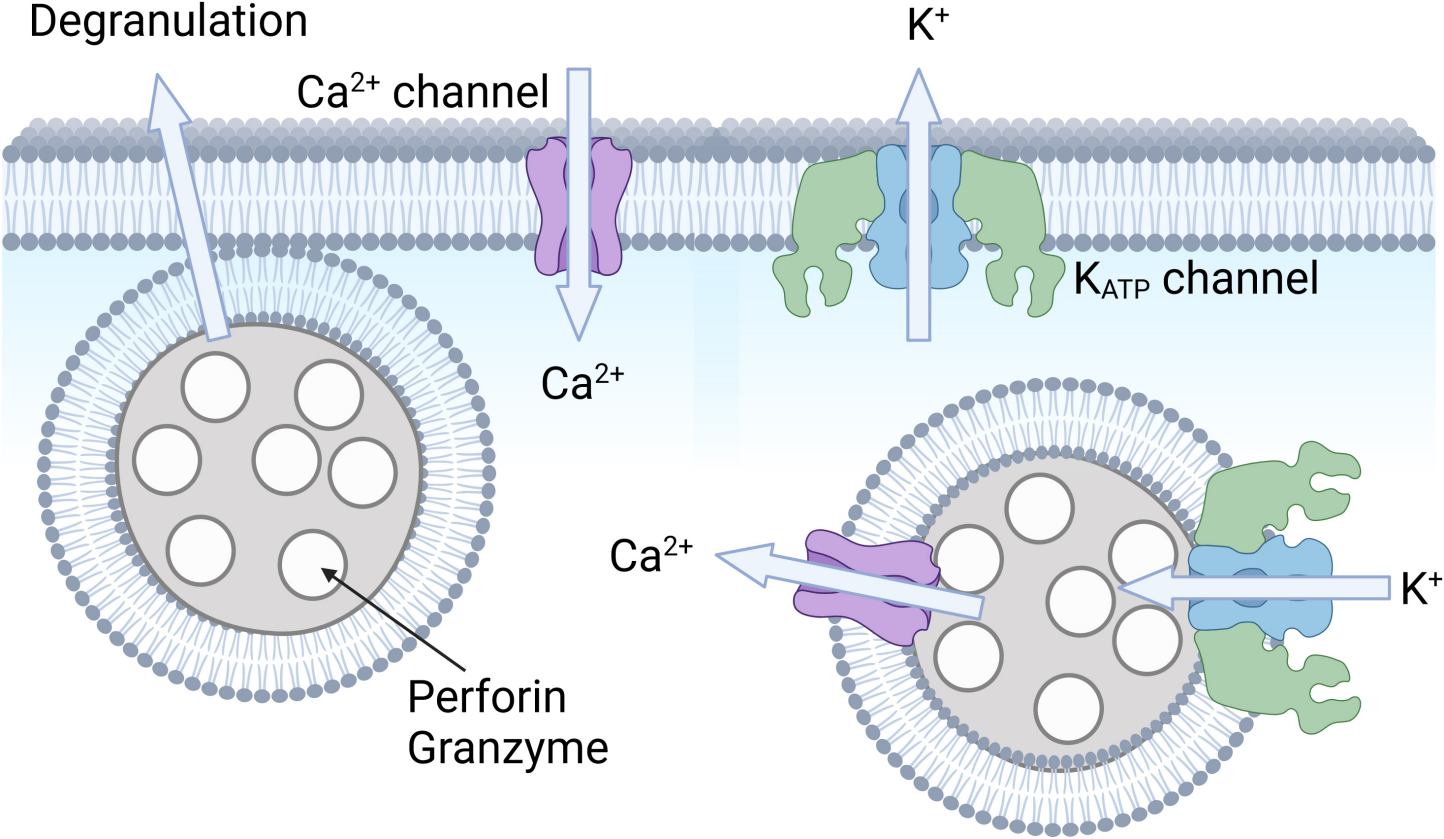
Hypothetical role of K_ATP_ channels in cytotoxic immune cells. Shown is an illustration of an immune cell, such as a CD8+ T cell or an NK cell. Intracellular vesicles (or granules) contain perforin and granzyme, which are released upon cell stimulation. It is possible that K_ATP_ channels and Ca^2+^ signaling may be involved in this degranulation process. It is also possible that K_ATP_ channels allow K^+^ influx to balance charge upon Ca^2+^ release from these lysosomal compartments. Created with BioRender.com.

K_ATP_ channels may affect immune cells in several ways. An established role of K_ATP_ channels in several cell types is to link channel activity to secretory events. The best described is the role of K_ATP_ channels in insulin secretion in the pancreatic β-cell, where K_ATP_ channel activity is blocked by high plasma glucose, leading to increased cellular excitability, elevated intracellular Ca^2+^, and exocytosis of insulin-containing granules (**Figure 1**). Under the same conditions, while K_ATP_ channel closure *stimulates* secretion in *β*-cells, it *inhibits* exocytosis of glucagon in pancreatic *α*-cells. The reason is that these two cell types have different underlying signaling pathways and express a different complement of other types of ion channels (55). K_ATP_ channels also affect secretory events in a number of other cell types. For example, they have a role both in the sympathetic and parasympathetic nervous system by respectively mediating secretion of norepinephrine (NE) and acetylcholine (18). In the central nervous system, there is evidence that K_ATP_ channels mediate the release of neurotransmitters, such as GABA from the substantia nigra (56, 57) and striatal dopamine release (58). K_ATP_ channels also regulate the exocytosis of ANP granules in the atria of the heart (59–62). The release events regulated by K_ATP_ channels are not limited to exocytosis. There is strong evidence for a role of K_ATP_ channels in the release of EDRF in smooth muscle (63), endothelin-1 from the endothelium (64, 65), musclin from skeletal muscle (66), ghrelin from the gastric mucosa (67), VEGF from pituitary folliculostellate cells (68) and the secretion of TNF and CXCL10 from pro-inflammatory macrophages markers (37). Thus, it is feasible that plasmalemmal K_ATP_ channels may contribute to exocytic release of perforin and granzyme in immune cells such as NK cells and CD8+ T cells (**Figure 5**) or participate in release of type I interferons, cytokines such as TFN-α, and interleukins such as IL-12 and IL-6. There is some evidence that K_ATP_ channels are linked to intracellular signaling pathways (possibly via membrane depolarization) that include NADPH oxidase (NOX2) activation and ROS production in pulmonary vascular endothelial cells (69). We therefore cannot exclude the possibility that K_ATP_ channels may affect signaling pathways involved in cytotoxicity of NK cells and other cytotoxic immune cells (70).

It may also be possible that K_ATP_ channels in intracellular organelles have a role to module immune cell function (**Figure 5**). In NK cells, for example, TRPML1-mediated Ca^2+^ release from acidic stores increases cytotoxicity by mediating human NK cell education (71). Ca^2+^ release from intracellular organelles is matched by K^+^ influx into the organelle to maintain electroneutrality (72). We previously found that some types of K_ATP_ channels localize to acidic compartments such as late endosomes and lysosomes (73). It is possible, therefore that K_ATP_ channels participate in Ca^2+^ release from acidic stores, thereby regulating immune cell function. This possibility remains to be explored.

Another attractive possibility is that K_ATP_ channels participate in regulating inflammasome function, particularly innate immune cells. The inflammasome is a multiprotein complex within most mammalian cells that plays a crucial role in immunity, particularly in the inflammatory response. The system is composed of pattern recognition receptors (PRRs) that detect pathogenic microorganisms and stress signals. These include NLRP3, NLRC4, and AIM2. The inflammasome is activated when these sensor proteins detect pathogenic bacteria, viruses, or other stress signals. The sensor proteins recruit ASC, which then recruits and activates caspase-1. Activated caspase-1 cleaves the pro-inflammatory cytokines pro-IL-1β and pro-IL-18 into their active forms, IL-1β and IL-18, which are then secreted by the cell to promote inflammation. Caspase-1 can also cleave gasdermin D and induce a form of programmed cell death known as pyroptosis, which helps to eliminate infected cells and alert neighboring cells to the presence of a pathogen. There is evidence that K^+^ plays a significant role in the activation of the inflammasome, particularly the NLRP3 inflammasome. A decrease in intracellular K^+^ concentration following the opening of K^+^ permeable channels is commonly thought to be a common signal for the activation of the NLRP3 inflammasome (74). K^+^ efflux from the cell appears to be important since various stimuli that activate the NLRP3 inflammasome (75). This K^+^ efflux affects the NLRP3 protein, possibly by causing a conformational change (74, 76), facilitating its oligomerization and subsequent recruitment of the adaptor protein ASC and the effector protein caspase-1.

Several K^+^ permeable channels such as P2X7 and *bona fide* K^+^ channels such as the two pore channels THIK‐1 (14) and TWIK2 (77) are increasingly implicated in inflammasome activation. Several lines of evidence indicate that K_ATP_ channels may also play a significant role in this process. The K_ATP_ channel blocker glibenclamide, for example, inhibits NLRP3 inflammasome-mediated IL-1β (and IL-8) secretion in several cell types, including human trophoblasts (78), primary human monocytes (33), human and murine macrophages (79–81), murine Schwann cells (82), and THP-1 cells (83). Similar effects of glibenclamide are also observed after intravenous injection of oleic acid to simulate pulmonary fat embolism (84), when granulocytes from human diabetic patients are exposed to *B. pseudomallei* (32), and the development of lipopolysaccharide- induced acute lung injury (ALI) (85). It is possible, however, that glibenclamide may affect targets other than K_ATP_ channels since the concentrations used in some of the *in vitro* studies exceeds that necessary to block K_ATP_ channels. Moreover, structural analogs of glibenclamide may retain effects on the inflammasome without having an effect on insulin secretion, suggesting that K_ATP_ channels are unaffected (86). Confoundingly, the K_ATP_ channel opener nicorandil, which is clinically used to prevent myocardial ischemia and angina symptoms through vasorelaxant effects on systemic and coronary vasculature, also inhibits inflammasome activation and pyroptosis (87–89). Nicorandil is a nitrate derivative of nicotinamide. Although it acts as a K_ATP_ channel opener, it also promotes smooth muscle via the nitrate moiety to cause relaxation and vasodilation by increasing cGMP activity. Since nitrates independently inhibit the inflammasome and pyroptosis (90, 91),it is presently unclear whether nicorandil affects these events due to an effect on K_ATP_ channels. As often is the case, the strongest evidence can be found in mouse genetics. Mice deficient of *Kcnj11* (Kir6.2) exhibited exacerbated liver injury, increased caspase-1 activity in the liver, and significantly elevated serum levels of IL-1β and TNF-α after treatment with LPS (92). Similarly, specific *Kcnj8* deletion in astrocytes increased NLRP3-mediated astrocytic pyroptosis in response to stress (93). Mice deficient in *Kcnj8* had elevated caspase-1 and IL-1β levels in the liver, whereas TNF-α, IL-6 and IFNγ were unchanged (94). Moreover, transfecting A549 cells with Kir6.1 led to inhibition of LPS-induced NLRP3 inflammasome activation. Thus, *Kcnj8* appears to be a negative regulator of the NLRP3 inflammasome in mice *in vivo* and in cultured cells *in vitro*. There is a contradiction since K^+^ efflux from the cell has been described to activate the NLRP3 inflammasome (75); yet the loss of K_ATP_ channel subunits activates the NLRP3 inflammasome. The underlying mechanisms are unclear. It is possible, for example, that the K_ATP_ channel subunits may affect the NLRP3 pathway via non-channel functions. These observations need to be investigated further.

## Perspectives and future directions

From examining mRNA expression levels in databases of immune cells, it becomes clear that the major K_ATP_ channel subunit genes expressed in mouse immune cells are *Kcnj8* and *Abcc9*. These subunits are constitutively expressed in NK cells and in NKT cells. The *Kcnj8* and *Abcc9* gene expression is markedly upregulated after infection in liver macrophages and in select populations of CD8+ T cells. Knocking out *Kcnj8* and *Abcc9* specifically in these cell types should reveal specific roles of K_ATP_ channels in immune surveillance and cancer immunity. Aside from direct roles in immune cells, *Kcnj8* (and possibly *Abcc9*) appear to have major roles as a “stabilizing” gene, functioning to survive inflammation and inflammation-induced stress. The cellular component appears to be extrahemapoietic and may include the coronary vasculature, but also brain microglia and vascular pericytes, which have immune functions. An important future direction is to generate mouse models deficient of *Kcnj8* (or possibly *Abcc9*) in these cell types and monitor their resistance to infection by MCMV or *Listeria*, or after infection with gram negative bacteria in the context of sepsis. Another important avenue of research is to determine the role of *Kcnj8* in antitumor immunity given the high expression of *Kcnj8* in macrophages, NK cells and CD8+ T cells and the importance of these cells for immune responses against cancer. Finally, although clinical studies with K_ATP_ channel blockers suggest a role for K_ATP_ channels in inflammation with humans, there are limitations of extrapolating mouse data to human immune function. It will be imperative to also investigate K_ATP_ channel gene expression in human immune cells. The K_ATP_ channel involvement in immunity can also potentially be further studied in patients (e.g. with diabetes) that are medicated with K_ATP_ channel modulating drugs.

## Data Availability

The original contributions presented in the study are included in the article/supplementary material. Further inquiries can be directed to the corresponding author.
